# Corrosion Resistance Study of Cyclocarboxypropyl Oleic Acid-Doped Polyaniline/Epoxy Composite Coatings

**DOI:** 10.3390/nano15181416

**Published:** 2025-09-15

**Authors:** Xinning Xu, Xiaofeng Li, Taihua Zhang, Wei Guo, Yan An, Tao Liu

**Affiliations:** College of Ocean Science and Engineering, Shanghai Maritime University, Shanghai 201306, China

**Keywords:** doped PANI, CCHOA, epoxy composite coating, corrosion resistance performance

## Abstract

Corrosion inhibitors can form dense, protective layers on metal surfaces, thereby preventing the penetration of corrosive media and ensuring the long-term safety of industrial equipment and energy facilities. Polyaniline (PANI), renowned for its excellent conductivity and redox activity, not only facilitates the formation of passivation layers on metals but also mitigates pitting corrosion. In this study, a novel doped PANI was synthesized through chemical oxidation using cyclic carboxypropyl oleic acid (CCHOA) as a dopant, and its anti-corrosion properties were evaluated by incorporation into epoxy resin coatings. Scanning electron microscopy (SEM), X-ray diffraction (XRD), and Fourier-transform infrared spectroscopy (FTIR) analyses confirmed that CCHOA-doped PANI produced a more uniform and compact microstructure with reduced agglomeration. The corrosion resistance and toughness of the epoxy coatings initially improved with increasing CCHOA content, but then slightly declined, which allowed us to determine the optimal doping level for PANI. The ideal concentration was found to be 0.5 mol/L in the epoxy resin matrix.

## 1. Introduction

Metal corrosion degrades material properties, which can shorten the lifespan of structures and endanger people’s safety. Therefore, developing new, effective corrosion protection methods is essential for safeguarding metals and enhancing equipment reliability [1]. Corrosion inhibitors are chemical agents that considerably slow down the rate of metal corrosion [2]. They create a protective film on metal surfaces through adsorption, preventing corrosive media from directly contacting the metal and thus reducing corrosion.

The addition of new chemical groups and structural modifications have continually improved the effectiveness of corrosion inhibitors. For example, attaching longer alkyl chains to imidazolium-based ionic liquids has significantly increased their corrosion inhibition efficiency for mild steel in acidic media, achieving 95.2% protection [3,4]. The adsorption capacity and inhibition performance of benzimidazole derivatives for copper in NaCl solution were notably enhanced (up to 98%) through modification with electron-donating amino and thiol groups [5].

Polyaniline (PANI) is an intrinsically conducting polymer with oxidizing properties, capable of adjustable metal adsorption and corrosion prevention through controlled doping with acids [6]. Compared to other conductive polymers, PANI is known for its unique redox reversibility, proton acid doping characteristics, and excellent thermal stability [7,8,9]. Different dopants significantly influence the corrosion inhibition performance of polyaniline [10,11].

Phytic acid (PA)-doped PANI has been used as a carrier for benzotriazole (BTA) corrosion inhibitors in epoxy coatings applied on Q235 steel. Electrochemical tests in 3.5 wt% NaCl solution showed that PA-PANI/BTA achieved an inhibition efficiency of 93.6%, notably higher than HCl-doped PANI/BTA (86.4%) [12]. This improvement was attributed to synergistic doping effects and increased hydrophobicity. Additionally, a mixed doping system using hydrochloric acid (HCl) and sodium dodecylbenzenesulfonate (SDBS) exhibited competitive doping behaviours in PANI. Research indicates that SDBS dominated the doping process because of its size and hydrophobic nature, leading to optimized performance [13]. Cyclocarboxypropyl oleic acid shows exceptional effectiveness in protecting metals from corrosion due to its unique dual-functional molecular structure and synergistic multi-mechanism actions: the long hydrophobic alkyl chain forms a physical barrier against corrosive media [14], while the cyclic carboxyl groups create stable chelation bonds through chemical adsorption on metal surfaces, enabling anodic passivation [15]. At the same time, it provides geometric coverage and interfacial charge modulation, significantly reducing corrosion rates (>90%) [16]. This paper prepares polyaniline doped with cyclohexylcarboxylic acid-modified oleic acid to combine the benefits of both components, delivering a new, highly effective corrosion inhibitor suitable for paint matrices and demonstrating excellent corrosion resistance.

## 2. Materials and Methods

### 2.1. Materials and Reagents

Aniline (analytical grade, Sinopharm Chemical Reagent Co., Ltd., Shanghai, China), ammonium persulfate (analytical grade, Shanghai Aladdin Biochemical Technology Co., Ltd., Shanghai, China), ethanol (analytical grade, Shandong Hongda Biotechnology Co., Ltd., Linyi, China), hydrochloric acid (analytical grade, Shanghai Chlor-Alkali Chemical Co., Ltd., Shanghai, China), cyclic carboxypropyl oleic acid (CCHOA, analytical grade, Guangzhou Daixun Trading Co., Ltd., Guangzhou, China), ethyl acetate (analytical grade, Yousuo Chemical Co., Ltd., Linyi, China), and deionized water (Dura ultrapure water system, Shanghai Hitech Instruments Co., Ltd. Shanghai, China) were used as received. Epoxy resin (E51, Yousuo Chemical Co., Ltd., Linyi, China) and polyetheramine curing agent (Yousuo Chemical Co., Ltd., Linyi, China) were used for coating preparation. Tinplate substrates (Baosteel Co., Ltd., Shanghai, China) were used as the metal substrate. Prior to coating, the substrates were polished with 600-grit sandpaper (Diaopai brand), degreased with acetone, rinsed with ethanol, and finally cleaned with deionized water.

### 2.2. Synthesis of CCHOA-Doped Polyaniline

CCHOA-doped polyaniline was synthesized using a chemical oxidation polymerization method. Five different concentrations of CCHOA solutions (0, 0.1, 0.3, 0.5, and 1 mol/L) were prepared by dissolving CCHOA in a mixed solvent of ethanol and water (7:3 volume ratio). Aniline solution (0.022 mol/L) was added dropwise to each CCHOA solution under vigorous stirring. The mixed solutions were then transferred to a low-temperature reactor and cooled to 3 °C. Simultaneously, ammonium persulfate (APS) solution (50 mL, with an equal molar concentration to aniline) was prepared and cooled to 3 °C. The APS solution was added dropwise to the reaction mixture at a rate of 1 mL/min using a constant-pressure dropping funnel. The reaction was maintained at 3 ± 0.5 °C for 24 h to ensure complete polymerisation. Afterwards, the product was separated by vacuum filtration and washed alternately with deionised water and ethanol (50 mL each) three times to remove unreacted monomers and impurities. The resulting product was dried in a vacuum oven at 60 ± 2 °C for 12 h and ground to pass through a 200-mesh sieve for further use.

### 2.3. Preparation of Epoxy Composite Coatings

Epoxy composite coatings were created by dispersing CCHOA-doped polyaniline (5 wt%) into 1.00 g of E51 epoxy resin and 1.00 g of polyetheramine curing agent, using a ratio of 100:35 for epoxy to curing agent, in a glass beaker. The mixture was stirred at 500 rpm for 5 min with a magnetic stirrer to produce a uniform composite slurry. This slurry was then drop-coated onto tinplate substrates that had been pre-treated with 400-grit sandpaper. The coated samples were cured in an oven at 65 ± 2 °C for 3 h, then naturally cooled to ambient temperature.

### 2.4. Characterization

Structural characterization was conducted using X-ray diffraction (XRD) with Cu Kα radiation (λ = 1.5406 Å). Measurements were carried out over a 2θ range of 10–80° at a scanning rate of 2° per minute. The surface morphology and microstructural features of the samples were further analyzed by field-emission scanning electron microscopy (FE-SEM, Zeiss Sigma 360, Oberkochen, Germany), operated at accelerating voltages of 15 kV and 5 kV, complemented by an energy-dispersive X-ray spectrometer (EDS).

Qualitative analysis was performed using a Fourier transform infrared spectrometer (FTIR, Nicolet iS5, Thermo Fisher Scientific, Waltham, MA, USA). Spectra were obtained in the mid-infrared range (4000–400 cm^−1^) in attenuated total reflection (ATR) mode, with a resolution of 4 cm^−1^ and an average of 32 scans. The baseline-corrected spectral data were analyzed using OMNIC software (Thermo Fisher Scientific, Waltham, MA, USA), and functional groups were identified by comparing the spectra to standard spectral databases.

The mechanical properties of the epoxy composite coatings were evaluated through tensile testing. The composite slurry was poured into dumbbell-shaped moulds (ASTM D638 standard) coated with a release agent (PTFE emulsion). The samples were initially cured at room temperature (25 ± 2 °C, 50 ± 5% relative humidity) for 24 h, then subjected to incremental curing in a drying oven (40 °C → 60 °C → 80 °C, 2 h at each step). After demolding, the edges of the samples were polished with fine sandpaper. Tensile tests were performed on a universal testing machine at a crosshead speed of 5 mm/min, with a gauge length of 45 mm. The stress–strain curves were recorded to assess the mechanical properties.

The corrosion resistance of the composite coatings was assessed using Electrochemical Impedance Spectroscopy (EIS). Tests were carried out in a three-electrode setup, with the coated tinplate as the working electrode, Ag/AgCl (3 M KCl) as the reference electrode, and a platinum sheet as the counter electrode. EIS measurements took place in a 3.5% NaCl solution using an electrochemical workstation. A 10 mV amplitude sine wave was applied across a frequency range from 10^5^ to 10^−2^ Hz. Before testing, the open circuit potential (OCP) was monitored until it stabilized (fluctuation < 2 mV/min) to eliminate initial polarization effects.

The chemical stability of the composite coatings was tested through acid–base immersion experiments. The coated samples were submerged in 1 mol/L HCl solution (pH = 0.67) and 1 mol/L NaOH solution (pH = 13.7) for three days. After immersion, the samples were dried and weighed to determine the change in mass from their original state. These tests aimed to assess the corrosion resistance and chemical stability of the coatings in harsh chemical environments.

## 3. Results

### 3.1. Morphology and Structure Analysis

**Figure 1:**
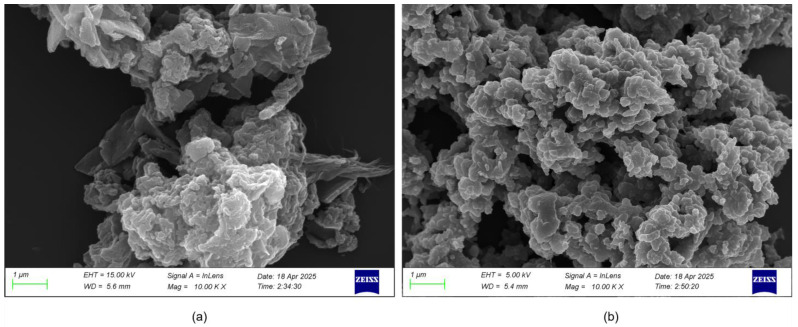
SEM images of PANI powder (**a**) without CCHOA and (**b**) doped with 0.5 mol/L CCHOA. The undoped PANI shows irregular agglomerates, whereas the doped sample exhibits a more compact and uniform morphology, reducing porosity and enhancing corrosion protection.

The SEM images show clear differences in microstructure between undoped PANI powder and 0.5 mol/L CCHOA-doped PANI powder. As shown in Figure 1, the undoped sample exhibits irregular, agglomerated structures with prominent block-like and flaky shapes, indicating significant particle clumping and a loose, uneven structure. This morphology results from strong intermolecular forces like hydrogen bonding and π-π stacking, which cause disordered molecular chain aggregation. In contrast, the 0.5 mol/L CCHOA-doped PANI powder features a more compact and even microstructure with less particle clumping and a finer, more regular morphology. The CCHOA molecules weaken intermolecular interactions by inserting between polyaniline chains, encouraging a more orderly molecular arrangement and creating a dense structure. This dense layer acts as a barrier, preventing corrosive media from penetrating, reducing contact with the interior of the material, and thus enhancing corrosion resistance.

**Figure 2:**
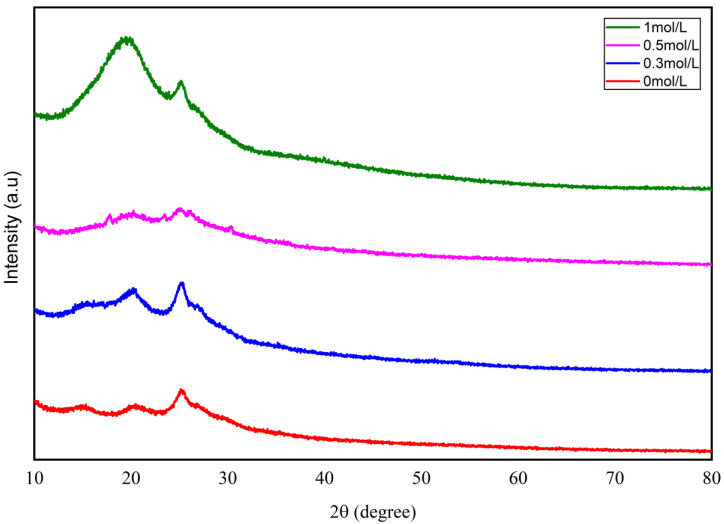
X-ray diffraction patterns of PANI powder doped with different concentrations of CCHOA. Increasing CCHOA doping alters crystallinity and induces partial lattice distortion, with 0.5 mol/L showing enhanced peak intensity at 26°, indicating improved local ordering.

The XRD analysis shows that the crystalline structure of PANI powder depends on the CCHOA doping level. As shown in Figure 2, for undoped polyaniline, a sharp diffraction peak appears at 2θ ≈ 26° (d ≈ 3.42 Å), likely caused by the weakly ordered arrangement of the (200) crystal plane of intrinsic polyaniline (Emeraldine Base, EB). Meanwhile, a broad peak below 10° (d ≈ 8.8 Å) reflects the disordered stacking of molecular chains, indicating the amorphous nature of EB. As the CCHOA doping concentration increases to 0.1 mol/L, new sharp peaks emerge at 2θ ≈ 21° (d ≈ 4.23 Å) and 26° (d ≈ 3.42 Å), with the 26° peak becoming much stronger. This suggests that hydrogen bonds form between the carboxyl groups of CCHOA and the imine nitrogen of polyaniline, promoting local molecular chain ordering. The long alkyl chains of CCHOA may also create layered structures through hydrophobic interactions, with the weak peak at low angles indicating a layer spacing of about 8–10 Å. At 20% concentration, the peak positions stay the same, but their intensity drops, and the full width at half maximum (FWHM) widens. This indicates a decrease in molecular chain order, likely caused by steric hindrance from excess CCHOA, reducing the crystalline phase stability. When doping reaches 0.5 mol/L, the 26° peak broadens and reaches maximum intensity but becomes less sharp, with the half-width increasing by approximately 30%. This reflects the formation of local aggregates of CCHOA molecules within the polyaniline matrix, which distort the lattice and expand amorphous regions. The 21° peak weakens at this level, probably due to competitive interactions between CCHOA and polyaniline, weakening the existing hydrogen bonds. These structural changes reveal a threshold effect of CCHOA doping: low concentrations (0.1 mol/L) improve molecular arrangement through interface self-assembly, while higher levels (0.5 mol/L) induce structural disorder due to molecular chain entanglement and phase separation.

**Figure 3:**
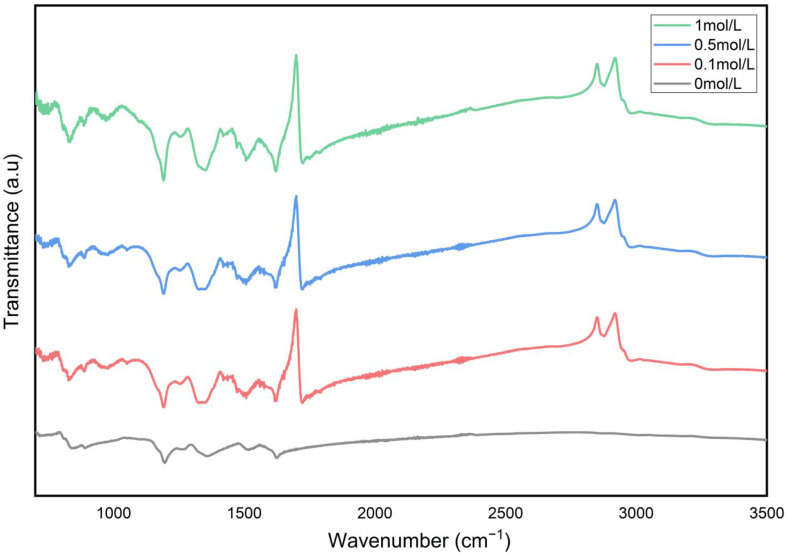
FTIR spectra of PANI powder doped with different concentrations of CCHOA. Characteristic C=O stretching peaks (~1700 cm^−1^) confirm successful doping, while CH_2_/CH_3_ stretching bands (~2850–2920 cm^−1^) indicate alkyl chain incorporation. The spectra show that CCHOA interacts with PANI mainly via hydrogen bonding without disrupting the conjugated backbone.

As shown in Figure 3, the FTIR analysis of CCHOA-doped PANI powder samples indicates that all samples have similar infrared spectra in the 400–2000 cm^−1^ range but display distinct features in the 2000–4000 cm^−1^ range due to varying CCHOA concentrations. A prominent absorption peak appears near 1700 cm^−1^ in all CCHOA-doped samples (0.1, 0.5, 1 mol/L), corresponding to the stretching vibration of the carboxyl group (C=O) in CCHOA. In contrast, the undoped (0 mol/L) sample lacks this peak, confirming it originates from CCHOA doping. Interestingly, the C=O peak does not significantly shift to lower wavenumbers (<1650 cm^−1^), suggesting that the interaction between CCHOA and polyaniline mainly occurs through hydrogen bonding or intermolecular forces, rather than complete protonation to form carboxylate salts. In the 2700–3000 cm^−1^ range, all CCHOA-doped samples show continuous weak absorption bands, attributed to the symmetric and asymmetric stretching vibrations of CH_2_ (~2850 cm^−1^) and CH_3_ (~2920 cm^−1^) in the long alkyl chains of CCHOA. The lower intensity of these bands may result from hydrophobic aggregation of alkyl chains that partially shield the infrared signal. Additionally, the characteristic peaks of the PANI backbone (e.g., C=C stretching vibrations of quinoid rings at 1560 cm^−1^ and 1480 cm^−1^, C-N stretching vibrations around 1300 cm^−1^) do not exhibit significant shifts after CCHOA doping, indicating that the introduction of CCHOA does not disrupt the conjugated backbone of polyaniline but may cause local hydrogen-bond network reconstruction. As the CCHOA concentration increases (from 0.1 to 1 mol/L), the intensity of the C=O peak gradually rises, indicating increased dopant loading. Meanwhile, the alkyl chain vibration peak remains relatively stable, suggesting that alkyl chains may form ordered hydrophobic regions through self-assembly, with their infrared response becoming saturated due to molecular orientation effects. These findings demonstrate that CCHOA effectively dopes PANI powder through polar interactions between its carboxyl groups and the polymer.

### 3.2. Mechanical Property Analysis

**Figure 4:**
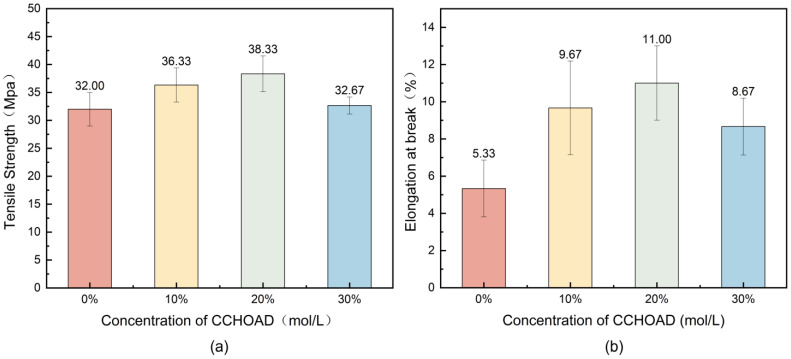
Mechanical properties of PANI/epoxy composite coatings doped with different concentrations of CCHOA: (**a**) Tensile strength peaks at 0.5 mol/L doping (38.3 MPa), about 20% higher than undoped samples. (**b**) Elongation at break also increases to 11%, confirming enhanced toughness at optimal doping.

The mechanical properties of CCHOA-doped PANI/epoxy composites show a non-linear relationship with CCHOA concentration, with tensile strength and elongation at break initially rising and then declining, as illustrated in Figure 4. When the CCHOA concentration reaches 0.5 mol/L, the tensile strength peaks at 38.33 MPa, representing a 20% increase compared to the undoped PANI/epoxy composite (32 MPa), while the elongation at break rises to 11% (compared to 5.33% for the undoped composite). This indicates that proper CCHOA doping significantly improves the mechanical properties of the composite. This improvement can be explained by the multi-scale synergistic interactions of CCHOA molecules with the PANI backbone within the epoxy matrix: carboxyl groups (–COOH) form hydrogen bonds with the imine nitrogen (-NH-) of polyaniline through protonation, strengthening interchain interactions. Meanwhile, long alkyl chains promote molecular chain alignment via van der Waals forces, reducing internal defects and preventing crack growth. Additionally, CCHOA acts as a plasticizer, balancing stiffness and flexibility, increasing chain segment mobility, and enhancing ductility. However, at a CCHOA concentration of 1 mol/L, both tensile strength and elongation at break decrease to 32.67 MPa and 8.67%, respectively, demonstrating the adverse effects of excessive CCHOA doping on the PANI/epoxy structure. These effects may stem from two factors: (1) excess CCHOA molecules cause phase separation within the epoxy matrix with PANI, forming hydrophobic microregions or clusters that raise stress concentration points and weaken interfacial bonds; (2) an overabundance of alkyl chains may disrupt the conjugated network of PANI dispersed in the epoxy resin, reducing molecular chain continuity and hindering effective crosslinking.

### 3.3. Corrosion Resistance Analysis

EIS analysis reveals that the impedance of CCHOA-doped PANI/epoxy composite coatings changes notably after immersion in 3.5 wt% NaCl solution, allowing for the assessment of coating failure and medium penetration into the substrate. The EIS results include Nyquist and Bode plots, with the high-frequency semicircle representing polarization resistance Rp. The diameter of this semicircle indicates the resistance between the PANI/epoxy composite film and the tinplate surface.

The analysis conducted after 1 h of immersion aims to evaluate the initial barrier effect of the coating before significant electrolyte penetration occurs, indicating its short-term protective capability. Conversely, the results obtained after longer immersion times are used to assess the durability of this protective effect and to observe how the coating structure evolves under continuous acid attack. This step-by-step comparison emphasizes both the early-stage inhibition efficiency and the long-term stability of the PANI/epoxy composite coatings.

**Figure 5:**
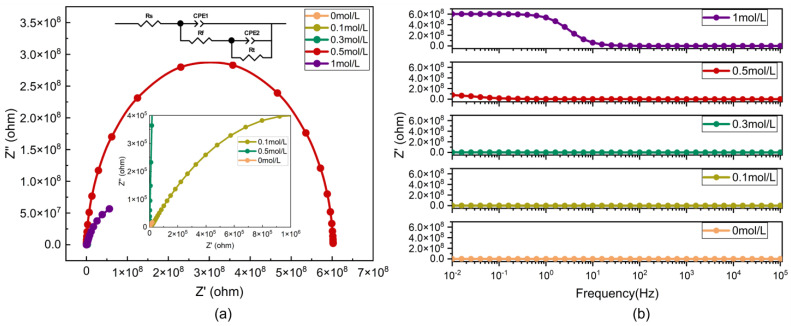
EIS spectra of PANI/epoxy composite coatings doped with different concentrations of CCHOA: (**a**) Nyquist plots and (**b**) Bode plots.

As shown in Figure 5a, it is clear that after 1 h of immersion, the sample doped with 0.5 mol/L CCHOA shows the largest capacitive diameter, indicating the best corrosion resistance. With increasing immersion time, the capacitive diameter decreases across all samples, indicating diminished coating protection and the infiltration of corrosive media into the substrate. From Figure 5b, it is evident that the impedance modulus and phase angle vary with frequency, with the impedance modulus of the 0.5 mol/L CCHOA-doped PANI/epoxy composite coating remaining highest after 1 h of immersion. Bode plot results show that the impedance modulus first increases and then gradually decreases with different composite epoxy coatings (notably a significant drop at 1 mol/L), suggesting that the 0.5 mol/L CCHOA-doped composite coating has superior corrosion resistance. Table 1 summarizes the equivalent circuit fitting parameters obtained from EIS measurements at different electrolyte concentrations. As shown in the table, both the film resistance (Rf) and the charge transfer resistance (Rt) increase markedly with the introduction of CCHOA, reaching the highest values at 0.5 mol/L. Specifically, Rf rises to 2.416 × 10^7^ Ω·cm^2^ and Rt increases to 5.71 × 10^8^ Ω·cm^2^ at this concentration, indicating the formation of a highly stable protective barrier. At 1 mol/L, however, both resistances decrease, suggesting that excessive CCHOA induces structural disorder and compromises long-term stability. These data strongly support the electrochemical evidence that 0.5 mol/L CCHOA provides the most effective corrosion protection.

At low concentrations (0–0.3 mol/L), the impedance modulus is low, with small semicircle diameters, reflecting low charge transfer resistance and easy penetration of corrosive media such as Cl^-^ and O_2_ through coating defects or pores, leading to localized electrochemical corrosion. This limitation stems from insufficient CCHOA content, which hampers the formation of a fully developed hydrogen bond crosslinking network between PANI chains within the epoxy matrix and results in weak hydrophobic self-assembly of alkyl chains. This causes porosity (>5%) and reduces the continuity of the conductivity network within the coating.

When CCHOA concentration increases to 0.5 mol/L, hydrogen bonding between CCHOA’s -COOH and the -NH- of PANI chains embedded in the epoxy matrix becomes significantly stronger, while alkyl chains promote molecular chain alignment via van der Waals forces, forming a highly ordered conductive network. However, further increasing CCHOA to 1 mol/L still yields an impedance modulus higher than that of low-concentration systems but shows a decreasing trend compared to 0.5 mol/L. This is due to local aggregation of excess CCHOA molecules within the PANI/epoxy composite, leading to phase separation and lattice distortion, which weakens the stability of the hydrogen bond network. Additionally, excess alkyl chains may cause steric hindrance, disrupting the conductivity network and forming hydrophobic microregions, which worsen internal stress and crack propagation within the coating.

**Figure 6:**
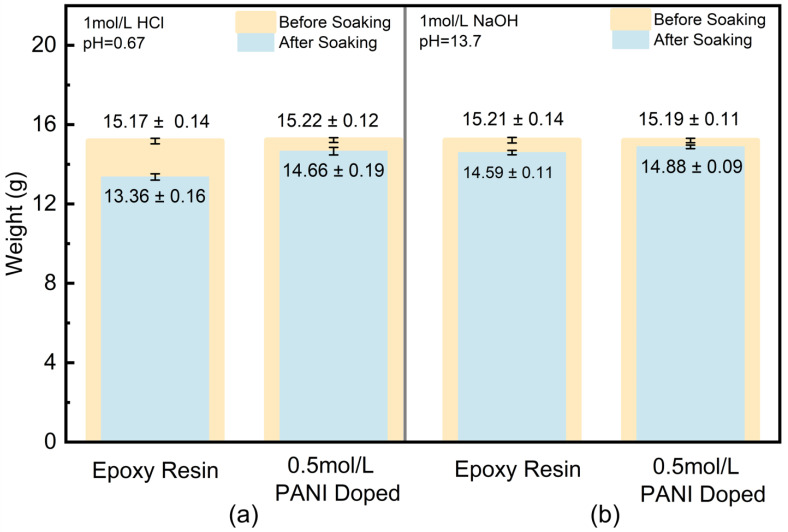
Weight loss of epoxy coatings and CCHOA-DOPED PANI composite coatings after 7-day immersion in (**a**) 1 mol/L HCl and (**b**) 1 mol/L NaOH. Doped coatings, particularly at 0.5 mol/L, show significantly lower mass loss, confirming improved acid and alkali resistance due to synergistic passivation and barrier effects.

As shown in Figure 6, in a 1 mol/L HCl solution, the weight loss rate of the pure epoxy resin coating is 11.9% (15.17 g → 13.36 g), while that of the 0.5 mol/L CCHOA-doped polyaniline-epoxy composite coating drops to 3.7% (15.22 g → 14.66 g), indicating a significant improvement in acid stability due to the addition of the composite material.

This enhancement is attributed to the dual effects of polyaniline: first, its redox activity promotes the formation of Fe_3_O_4_/γ-FeOOH composite passive films on the metal substrate, inhibiting corrosion caused by H+ and Cl- in acidic media; second, polyaniline molecular chains form a dense interpenetrating network with epoxy resin through π-π stacking, reducing porosity from 8% in pure epoxy resin coatings to below 3%, which effectively blocks the penetration of corrosive media. Additionally, the protonated form of polyaniline remains stable in acidic environments, facilitating charge transfer to inhibit localized galvanic corrosion and further reducing coating hydrolysis and swelling.

In a 1 mol/L NaOH solution, the weight loss rate of the pure epoxy resin coating is 4.6% (15.21 g → 14.59 g), while that of the 0.5 mol/L CCHOA-doped PANI/epoxy composite coating drops to only 2.0% (15.19 g → 14.88 g), demonstrating superior alkali resistance. This can be attributed to the structural stability of PANI dispersed in the epoxy resin under alkaline conditions: although strong alkaline environments may cause partial dedoping of polyaniline, its conjugated backbone remains tightly bonded to the epoxy resin through hydrogen bonds, maintaining the coating’s mechanical integrity. Additionally, the long-range π-electron system of polyaniline can neutralize chemical attacks from OH-, which delays the alkaline hydrolysis of the epoxy resin molecular chains.

## 4. Discussion

The findings of this study highlight the significant impact of cyclocarboxypropyl oleic acid (CCHOA) doping levels on the structure and performance of polyaniline (PANI)-based epoxy composite coatings. Microstructural analysis clearly demonstrated that adding CCHOA effectively changed the morphology of PANI powder, reducing agglomeration and promoting a denser, more uniform polymer matrix. This nanostructural improvement is essential because it influences the coating’s ability to block corrosive agents like chloride ions and oxygen. Importantly, this morphological enhancement is closely connected to the observed improvements in mechanical and corrosion resistance properties.

Mechanical testing revealed a concentration-dependent trend, where adding CCHOA initially enhanced tensile strength and elongation at break, peaking at 0.5 mol/L doping. This effect can be attributed to improved polymer chain interactions and plasticization effects caused by moderate dopant levels, which not only strengthen the mechanical properties but also boost flexibility and toughness. These improvements help the coating withstand mechanical stresses and environmental damage. However, beyond this concentration, the physical and chemical stability of the composite coating was compromised. Excessive doping (1 mol/L) led to dopant aggregation and phase separation, as shown by spectral and diffraction analyses that indicated the formation of structural defects and disruption of the conjugated polymer backbone. This deterioration negatively impacted charge transport within the polymer and the integrity of the coating network, resulting in decreased mechanical strength and reduced corrosion protection.

The electrochemical results further support this interpretation, demonstrating that coatings doped at the optimal CCHOA concentration exhibited the highest impedance and lowest corrosion rates in simulated acidic and alkaline environments. These findings indicate that moderate CCHOA doping not only enhances physical barrier properties but also improves electrochemical passivation by promoting the formation of stable, protective oxide layers on metal surfaces. The hydrophobicity provided by CCHOA molecules also reduces water absorption and ionic diffusion, helping to decrease corrosion. However, at higher dopant levels, reduced polymer integrity and increased defects likely create pathways for corrosive agents, weakening the protective coating.

Compared to pure epoxy coatings, the CCHOA-doped PANI composites showed notable improvements in both mechanical resilience and corrosion resistance. This demonstrates a synergistic effect where the conductive polymer network enhances the coating’s redox ability to form passivating films, while the epoxy matrix offers mechanical support and adhesion. The distinctive redox-active properties of PANI, boosted by proper doping, allow for dynamic protection that exceeds simple barrier blocking. Specifically, at the metal/coating interface, the oxidation–reduction activity of PANI facilitates the formation of a stable Fe_3_O_4_/γ-FeOOH passive film, which suppresses localized corrosion, while CCHOA contributes to a dense interpenetrating network with the epoxy, lowering porosity from ~8% in pure epoxy to <3% in the doped system. In addition, hydrogen bonding and π–π stacking interactions ensure strong interfacial adhesion, while the conjugated backbone of PANI maintains structural stability. This dual mechanism creates a reliable long-term protection system in which barrier effects and passivation effects act synergistically. This multi-layered mechanism provides more reliable long-term protection under harsh conditions.

However, the short-term laboratory tests used in this study do not fully simulate long-term environmental exposures like those in marine or industrial atmospheres. Durability against UV radiation, cyclic temperature and humidity changes, and mechanical wear still need to be studied. Additionally, the molecular interactions that control dopant distribution and bonding in the PANI matrix are not yet fully understood. Using molecular dynamics simulations combined with advanced spectroscopic techniques could provide insights into the doping process and interfacial chemistry at the atomic level.

Future work may also focus on incorporating nanoscale fillers like graphene or MXenes to develop multiscale composite coatings that harness synergistic mechanical improvements and enhanced ionic barrier properties [17]. Stimuli-responsive and self-healing coating systems that include doped PANI also show promise, aiming to extend service life and reliability. Ultimately, optimizing the balance between performance, environmental impact, and manufacturing costs will be essential for advancing the practical use of these materials in marine engineering, the petrochemical industry, and other harsh environments.

## 5. Conclusions

In this study, polyaniline powders doped with different molar concentrations of cyclocarboxypropyl oleic acid (CCHOA) were synthesized and incorporated into epoxy resin to create composite coatings. The relationship between dopant concentration and the coatings’ mechanical and corrosion resistance properties was thoroughly examined. The main conclusions are as follows:

The successful doping of CCHOA into the PANI matrix was confirmed by SEM, FTIR, and XRD analyses, which also demonstrated that CCHOA doping modifies the PANI microstructure, resulting in denser and more uniform morphologies that reduce particle aggregation.

Mechanical tests, acid–base immersion experiments, and electrochemical impedance spectroscopy (EIS) indicated that the properties of the composite coatings initially improve and then slightly decline as CCHOA concentration increases. The optimal doping concentration was identified as 0.5 mol/L, where tensile strength and elongation at break reached their highest levels (38.33 MPa and 11%, respectively), and the coating showed enhanced corrosion resistance compared to pure epoxy resin coatings.

At higher doping concentrations (1 mol/L), the coating’s properties deteriorate due to dopant aggregation and defect formation, which compromise the polymer chain integrity and protective barrier performance.

Compared to pure epoxy coatings, the CCHOA-doped PANI/epoxy composite coatings exhibited significantly improved corrosion resistance and mechanical toughness, underscoring their strong potential for industrial corrosion protection applications [18].

This study provides a foundation for further development of multifunctional, conductive polymer-based composite coatings with adjustable properties for metal protection. Future research should focus on assessing long-term durability, performing advanced molecular-level characterization, incorporating nanofillers for multi-scale enhancements, and exploring eco-friendly materials for sustainable commercialization.

## Figures and Tables

**Table 1:** Equivalent circuit fitting parameters obtained from EIS measurements at different electrolyte concentrations.

	0 mol/L	0.1 mol/L	0.3 mol/L	0.5 mol/L	1 mol/L
Rs (Solution resistance)/Ω cm^2^	6.122	1310	186.5	1588	61.79
CPE1 (Constant phase element 1)/F cm^−2^	1.116 × 10^−4^	1.089 × 10^−10^	4.953 × 10^−10^	6.800 × 10^−11^	1.036 × 10^−10^
n1 (Exponent of CPE1)	0.7556	1	0.8968	1	0.9775
Rf (Film resistance)/Ω cm^2^	1.159 × 10^4^	1.205 × 10^4^	5.600 × 10^4^	2.416 × 10^7^	9.386 × 10^5^
CPE2 (Constant phase element 2)/F cm^−2^	2.333 × 10^−4^	4.732 × 10^−7^	5.698 × 10^−7^	1.368 × 10^−11^	4.040 × 10^−8^
n2 (Exponent of CPE2)	0.8663	0.4778	0.6441	1	0.7773
Rt (Charge transfer resistance)/Ω cm^2^	4.060 × 10^4^	2.030 × 10^6^	2.730 × 10^6^	5.710 × 10^8^	1.810 × 10^8^

## Data Availability

The data presented in this study are available upon request.

## Abbreviations

The following abbreviations are used in this manuscript:

CCHOA	Cyclocarboxypropyl Oleic Acid
EB	Emeraldine Base
EIS	Electrochemical Impedance Spectroscopy
ES	Emeraldine Salt
FTIR	Fourier Transform Infrared Spectroscopy
MD	Molecular Dynamics
MXene	Two-dimensional transition metal carbides/nitrides
PANI	Polyaniline
SEM	Scanning Electron Microscopy
TEM	Transmission Electron Microscopy
XRD	X-ray Diffraction
